# Simplification of Low-Cycle Creep–Fatigue Load Spectrum of Combustion Chamber and Life Assessment for Feature Simulation Specimens

**DOI:** 10.3390/ma19030620

**Published:** 2026-02-05

**Authors:** Dingnan Cheng, Honghua Zhao, Qiang Zhang, Minmin Chen, Hao Zhao, Cheng Hou

**Affiliations:** 1 AECC Hunan Aviation Powerplant Research Institute, Zhuzhou 412002, China; 2 National Key Laboratory of Science and Technology on Aero-Engine Aero-Thermodynamics, Research Institute of Aero-Engine, Beihang University, Beijing 100191, China; 3 School of Mechanical Engineering, Xi’an Jiaotong University, Xi’an 710049, China

**Keywords:** GH3230 superalloy, original load spectrum, simplified load spectrum, creep–fatigue, life prediction

## Abstract

**Highlights:**

**What are the main findings?**

- A simplified load spectrum for low-cycle creep–fatigue of combustion chambers was developed with experimental validation.
- Different combustion chamber feature simulation specimens were tested under original and simplified load spectrums.
- The low-cycle creep–fatigue life of the combustion chamber feature simulation specimens was predicted.

**What are the implications of the main findings?**

- The simplified load spectrum yielded a 6.13% average life error compared to the original spectrum, with both results lying within the double dispersion band.
- The experimental results of the flat specimens with single or multiple holes were both within the double dispersion band of the predicted results.
- Internal cooling gas flow reduced temperatures near film-cooling holes in tubular specimens, enhancing their low-cycle creep–fatigue life.

**Abstract:**

Based on the damage equivalence principle, simplification of the low-cycle creep–fatigue original load spectrum of a combustion chamber under multi-stage flight conditions, such as low speed, takeoff, climb, and cruise states, and experimental verification were carried out in this study. The low-cycle creep–fatigue life of the combustion chamber feature simulation specimens was predicted. The results showed that compared with the original load spectrum, the simplified load spectrum had an average life error of 6.13% in the low-cycle creep–fatigue tests of flat-plate specimens with a single hole. The simplified load spectrum test results and the original load spectrum test results were both within the double dispersion band of their average values. The low-cycle creep–fatigue test results of the flat specimens with single or multiple holes were both within the double dispersion band of the predicted results, while the test results of circular tube specimens with multiple holes were basically within the fourfold dispersion band of the predicted results. In addition, after passing cooling gas inside the circular tube test specimens with multiple holes, the temperature near the gas film holes was reduced, thereby improving their low-cycle creep–fatigue test life.

## 1. Introduction

A critical hot-end component of aeroengines, the combustion chamber operates for a long time in high-temperature environments, and its structural integrity and durability directly affect the safety and reliability of the engine [1,2,3]. However, during long-term service of the engine, the combustion chamber will be continuously subjected to thermomechanical loads, gradually accumulating damage caused by the interaction of creep and fatigue, which may lead to component failure and even engine failure in severe cases [4,5,6,7]. Therefore, it is essential to investigate the creep–fatigue failure behaviors of combustion chamber materials to ensure the safe and stable operation of combustion chambers.

There have been many studies on the creep–fatigue behaviors of combustion chamber materials [5,6,7,8,9,10]. Zhang et al. [5] studied the thermal fatigue crack initiation and propagation behaviors of GH3230 superalloy sheet specimens with V-notches and found that the thermal fatigue crack initiation life gradually decreased with increased peak temperature. Hou et al. [6,7] studied the effect of U-notches on creep behaviors of GH3230 superalloy at 900 °C and 1000 °C. The results showed that notches exhibited a creep life-enhancing effect on GH3230 superalloy under the same net stress level. Both stress concentration factor and net stress collectively determined the notch life enhancement factor. Zhao et al. [8] investigated the fatigue behaviors of Hastelloy X superalloy at 750 °C, characterizing the fatigue properties of small punch fatigue specimens through the relationship between load, cycle count, and displacement. Esmaeilzadeh et al. [10] proposed an improved texture evolution prediction model for the thermomechanical fatigue behavior of Hastelloy X superalloy, demonstrating good agreement with experimental results.

Although the above studies have deepened the understanding of material behaviors, in the actual testing and evaluation of combustion chambers, if creep–fatigue tests are carried out completely according to real service conditions, it will be time-consuming and costly, which is not conducive to the rapid development and iteration of engines [10,11]. Therefore, it is of significant engineering application value to conduct research on simplified methods for low-cycle creep–fatigue load spectra applicable to combustion chambers, which can effectively shorten test cycles and reducing development costs [12,13,14]. Load spectrum simplification and equivalent methods can be divided into three categories: (1) damage-based methods [15]; (2) energy/frequency-based methods [16]; and (3) multifeature fusion methods [17]. Zheng et al. [17] proposed a new method for editing the multiaxial load spectrum by combining the characteristics of damage and energy domain. By synthesizing the uniaxial spectrum, analyzing the energy and damage distribution, identifying the key segments, and reconstructing the half sine wave, significant compression of the spectrum duration was achieved, maintaining a load equivalent to the original spectrum. Chen et al. [18] proposed a simplified method of von Mises’s equivalent stress spectrum with symbol correction, which can make multiaxial stress spectra equivalent to uniaxial spectra for fatigue life prediction. The results showed that this method was more accurate than the classical equivalent spectrum and uniaxial spectrum, and was helpful in solving the engineering application problems of multiaxial fatigue theory. In terms of simplifying load spectra and accelerating experiments, some scholars have conducted beneficial explorations [19,20,21,22]. Jiang et al. [19] studied the effect of creep–fatigue load sequence within the load spectrum on life and developed a life prediction method based on the loading sequence effect. It was found that the life prediction results were consistent with the creep–fatigue test results within the double deviation band. He et al. [20,21] constructed an average spectrum and severe spectrum for fatigue testing of LC4 aluminum alloy specimens based on analysis of normal load distribution. The results indicated that the average lifetime under the mean spectrum was 1.5 times that under the severe spectrum. Li et al. [22] presented two kinds of load spectrum simplification approach based on a statistical consistent fatigue damage model: one method was to simplify the original multi-level load spectra to spectral loads with fewer levels, while the other converted the life distribution under multi-level load spectra into that of a constant-amplitude spectrum according to a certain principle. The results indicated that the equivalent damage simplification method for multi-level load spectra was feasible, ensuring that the damage per loading block before and after simplification was statistically equivalent.

However, a systematic simplification method and experimental verification are still needed for the low-cycle creep–fatigue load spectrum of combustion chambers of aeroengines under flight conditions. Therefore, based on the damage equivalence principle, a simplification of the low-cycle creep–fatigue original load spectrum of a combustion chamber under multi-stage flight conditions was conducted with a complex multi-stage spectrum equivalent to a simple spectrum. Then, the simplification method was experimentally validated using combustion chamber feature simulation specimens. Finally, the low-cycle creep–fatigue life of the feature simulation specimens with holes under complex multiaxial stress states was predicted by considering hole characteristics.

## 2. Materials and Methods

### 2.1. GH3230 Superalloy

GH3230 superalloy, a high-performance solution-strengthened nickel base superalloy, is known for its excellent high-temperature strength, outstanding oxidation resistance, and corrosion resistance. It is the preferred material for key high-temperature components in aerospace, such as the combustion chamber [5,6,7]. According to the *China Superalloys Handbook* [23], the chemical properties of combustion chamber material GH3230 superalloy are shown in Table 1, and its mechanical and thermal properties are shown in Table 2. The density of GH3230 superalloy is 8.934 g/mm^3^ and its creep endurance performance are shown in Table 3.

### 2.2. Larson–Miller Parameter (LMP) Method

The comprehensive curve equation of the thermal strength parameters of GH3230 high-temperature alloy is shown in Equation (1).

(1) $$\mathrm{lg}\left(\sigma \right)=a_{0}+a_{1}P+a_{2}P^{2}+a_{3}P^{3}$$

(2) $$P=\mathrm{lg}\left(t\right)+b\left(T+C\right)$$

**Figure 1:**
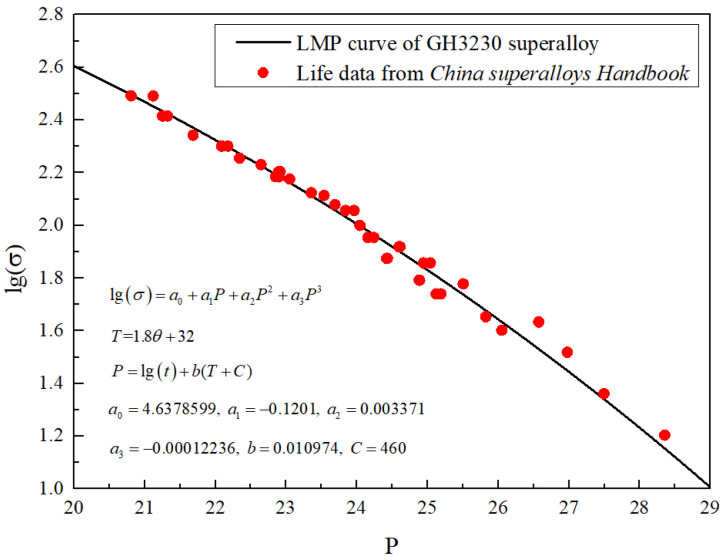
LMP fitting curve of GH3230 superalloy.

Here, *T* = 1.8*θ* + 32. *T* is the Fahrenheit temperature, *θ* is the Celsius temperature, and *σ* is the creep stress. *a*_0_, *a*_1_, *a*_2_, *a*_3_, *b*, and *C* are the coefficients for the Larson–Miller parameter (LMP) equation. Referring to the data in Table 3, the coefficients *a*_0_, *a*_1_, *a*_2_, *a*_3_, *b*, and *C* can be obtained from the fitting curve shown in Figure 1, with *a*_0_ = 4.6378599, *a*_1_ = −0.1201, *a*_2_ = 0.003371, *a*_3_ = −0.00012236, *b* = 0.010974, and *C* = 460, respectively.

Therefore, when the service temperature θ and stress σ are known, the creep endurance life t of GH3230 superalloy can be obtained according to Equations (1) and (2).

### 2.3. Creep–Fatigue Damage Assessment of GH3230 Superalloy Under Multi-Stage Flight Conditions

In order to evaluate the creep–fatigue damage to the GH3230 superalloy under multi-stage flight conditions, such as low speed, takeoff, climb, and cruise states, the damage equivalence principle is employed to calculate the creep damage for each stage. Then, according to the linear cumulative damage principle, the creep damage *D*_creep_ and fatigue damage *D*_fatigue_ to the GH3230 superalloy under multi-stage flight conditions in each cycle can be calculated. Equations (3) and (4) and the total creep–fatigue damage *D* in each flight cycle can be obtained as Equation (5):

(3) $$\begin{array}{l} D_{\mathrm{creep}}=\sum D_{i}=D_{\mathrm{slow}\;\mathrm{speed}}+D_{\mathrm{takeoff}}+D_{\mathrm{climb}}+D_{\mathrm{cruise}}\\ =\sum \frac{t_{i}}{T_{i}}=\frac{t_{\mathrm{slow}\;\mathrm{speed}}}{T_{\mathrm{slow}\;\mathrm{speed}}}+\frac{t_{\mathrm{takeoff}}}{T_{\mathrm{takeoff}}}+\frac{t_{\mathrm{climb}}}{T_{\mathrm{climb}}}+\frac{t_{\mathrm{cruise}}}{T_{\mathrm{cruise}}} \end{array}$$

(4) $$D_{\mathrm{fatigue}}=\frac{1}{N_{f}}$$

(5) $$D_{\mathrm{totel}}=D_{\mathrm{creep}}+D_{\mathrm{fatigue}}$$

where *i* represents one of the flight conditions, such as low speed, takeoff, climb, or cruise states. *D_i_* is the damage of the corresponding flight condition, *t_i_* is the loading time under the corresponding flight condition, and *T_i_* is the creep life calculated from Equation (1) for the corresponding flight condition. *N_f_* is the fatigue life of the material during each flight cycle, which only includes temperature and stress loading and unloading processes without the creep processes. *N_f_* can be obtained through thermomechanical fatigue experiments. Therefore, when the total creep–fatigue damage *D*_totel_ accumulates to 1, the material or structure will undergo cracking failure.

As a typical multiaxial stress structure, a combustion chamber’s film cooling holes can effectively reduce its temperature. However the local stress concentration caused by holes will shorten the creep–fatigue life of the combustion chamber. According to the experimental results from *Structural strength of film cooling holes in nickel-based single-crystal turbine blades* [24], the creep–fatigue life of superalloy specimens will be reduced by about 50% due to the presence of holes under the same nominal stress level. Therefore, in this paper, the creep–fatigue life of GH3230 superalloy specimens with holes can be considered half that of specimens without holes under the same nominal stress.

### 2.4. Simplified Load Spectrum Compilation Based on the Damage Equivalence Principle

**Figure 2:**
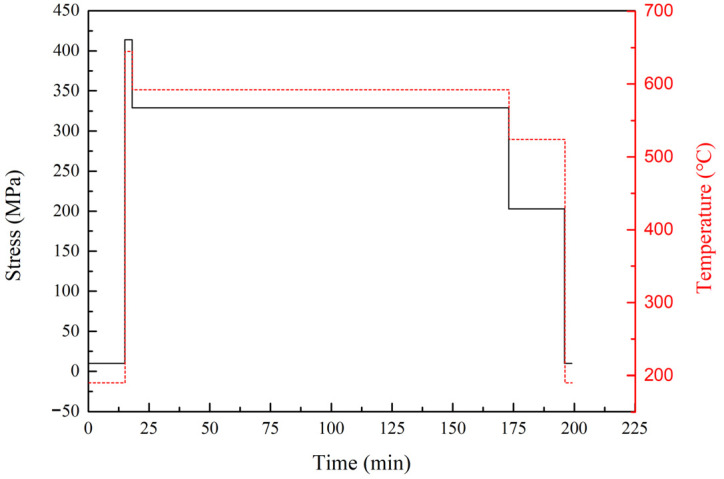
Original load spectrum for dangerous combustion chamber position in single flight mission cycle.

According to the service conditions of a combustion chamber under flight mission from the AECC Hunan Aviation Powerplant Research Institute, the original load spectrum for a dangerous combustion chamber position can be determined as shown in Table 4 and Figure 2. The flight mission cycle can be divided into four service conditions: low speed, takeoff, climb, and cruise conditions. A single flight mission cycle is 199 min, with a highest temperature of 645 °C and maximum stress of 414 MPa during the takeoff condition.

**Figure 3:**
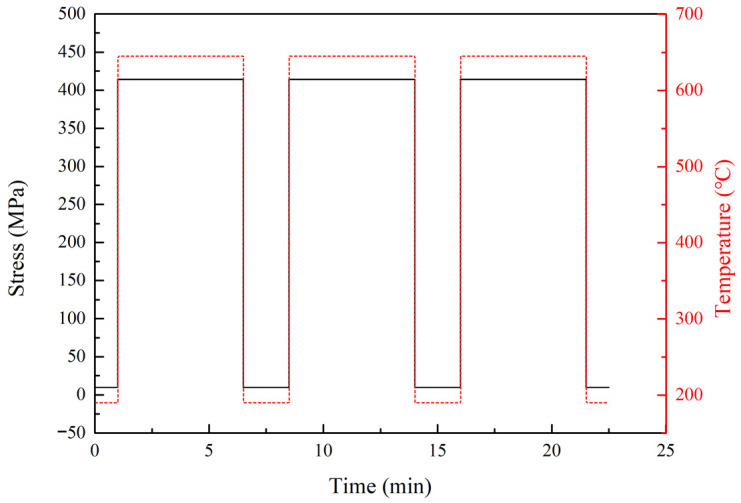
Simplified load spectrum based on the damage equivalence principle.

According to the LMP method, the total creep damage during a single flight mission cycle can be calculated and obtained as 1.555 × 10^−3^, as shown in Table 5. Based on the damage equivalence principle, the low-cycle creep–fatigue damage during a single flight mission cycle can be equivalently converted into simple low-cycle creep–fatigue damage during the low-speed and takeoff conditions. Since the fatigue damage during a single flight mission cycle before and after an equivalent conversion is equal, only the creep damage during a single flight mission cycle is equivalently converted. After equivalent conversion, the creep damage during a single flight mission cycle is equivalent to the 5.5 min service damage during takeoff condition. Therefore, the simplified load spectrum based on the damage equivalence principle is shown in Figure 3.

## 3. Experiments

### 3.1. Design of Feature Simulation Specimens

**Figure 4:**
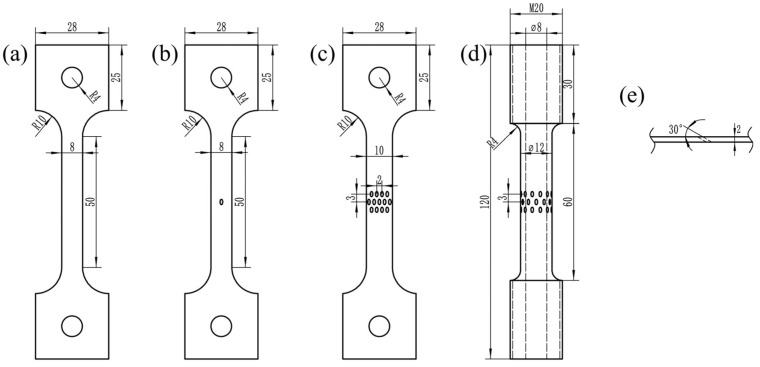
Feature simulation specimens. (**a**) Smooth flat-plate specimen; (**b**) flat specimen with single hole; (**c**) flat specimen with multiple holes; (**d**) circular tube specimen with multiple holes; (**e**) size of hole.

The design of feature simulation specimens of combustion chambers can be divided into four types, as shown in Figure 4: (a) smooth flat-plate specimen; (b) flat specimen with single hole; (c) flat specimen with multiple holes; and (d) circular tube specimen with multiple holes. To ensure that the hole characteristics of the feature simulation specimens are consistent with the characteristics of the real combustion chamber gas film holes, the hole sizes of the feature simulation specimens are designed as follows. The thickness of the smooth flat-plate specimen is 2 mm; the hole diameter and thickness of the flat specimen with a single hole are 1 mm and 2 mm, respectively, with an angle of 30° between the hole’s center axis and the specimen plane (as shown in Figure 4e), and the hole’s long-axis direction is consistent with the specimen tensile direction; and the hole diameter and thickness of the flat specimen with multiple holes is 1 mm and 2 mm, respectively, with three rows of inclined holes in the center. The angle between the holes’ center axis and the specimen plane is 30°, and the holes’ long-axis direction is consistent with the specimen tensile direction, with longitudinal spacing between the holes of 3 mm and horizontal spacing between the holes of 2 mm; the hole diameter and wall thickness of the circular tube specimen with multiple holes are 1 mm and 2 mm, respectively, with three rows of inclined holes in the center. The inclination angle of the holes is 30° and the holes’ long axis direction is consistent with the specimen tensile direction, with longitudinal spacing between the holes of 3 mm. There are 12 holes in one circle of the circular tube specimen, with a 30° central angle between adjacent holes. All sample processing is carried out on GH3230 superalloy cylindrical blocks that have been hot-rolled and solution-treated, with sampling in the LT direction. The surface roughness Ra of the processed sample is less than 1.6 μm, and the gas film holes of the sample are formed by laser drilling. The number of low-cycle creep–fatigue test specimens is shown in Table 6.

### 3.2. Low-Cycle Creep–Fatigue Tests

**Figure 5:**
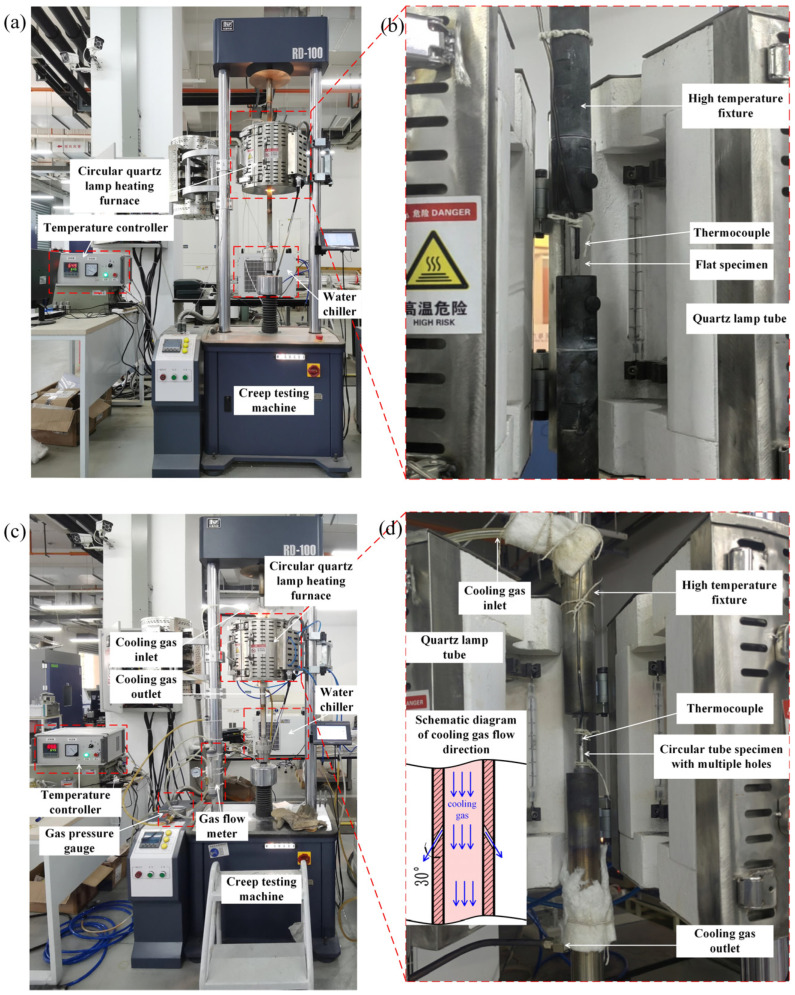
Load spectrum verification tests. (**a**,**b**) Flat specimens with single or multiple holes subjected to original load spectrum and simplified load spectrum; (**c**,**d**) circular tube specimens with multiple holes subjected to simplified load spectrum.

The low-cycle creep–fatigue tests were carried out using a creep testing machine (RD-100, Changchun Kexin Experimental Instrument Co., Ltd., Changchun, China) with a circular quartz lamp radiation heating furnace, as shown in Figure 5. The thermocouple was placed at the center of the sample and fixed in contact with it using high-temperature resistant rope, as shown in Figure 5b,d. The specimens can be heated by the circular quartz lamp radiation heating furnace with a heating/cooling rate of 20 °C/s and temperature control error after stabilization of less than 1 °C. The heating furnace was cooled by a water cooler to prevent overheating of the copper electrode at the connection end of the quartz lamp tube.

**Figure 6:**
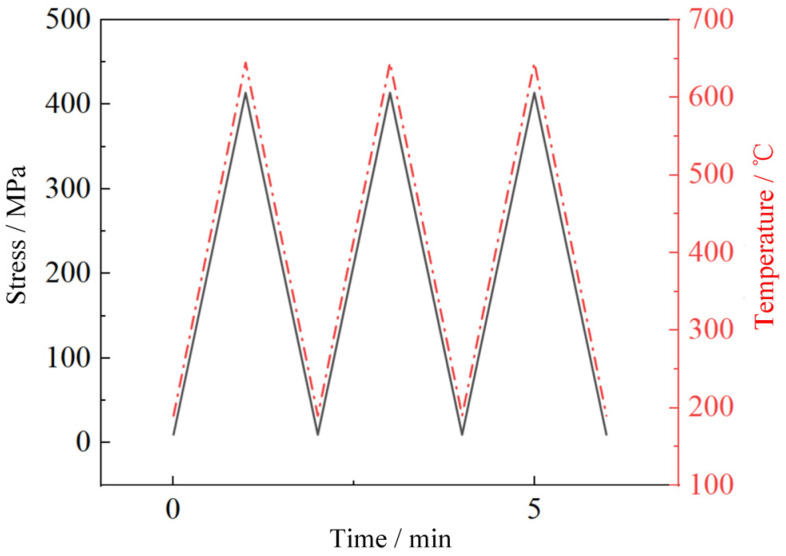
Thermomechanical fatigue test spectrum of smooth flat-plate specimens.

The original load spectrum and simplified load spectrum of the low-cycle creep–fatigue test are shown in Figure 2 and Figure 3, respectively. The smooth flat-plate specimens were used to conduct the thermomechanical fatigue tests, which only included the loading and unloading processes of temperature and stress, with a maximum temperature of 645 °C and a maximum stress of 414 MPa, as shown in Figure 6. The low-cycle creep–fatigue tests of the flat specimens with a single hole or multiple holes are shown in Figure 5a,b. After clamping the test specimen and thermocouple, the circular quartz lamp heating furnace was closed and the tests started.

The low-cycle creep–fatigue tests of the circular tube specimens with multiple holes are shown in Figure 5c,d. During the tests, a cooling gas was introduced into the center of the circular tube specimens with multiple holes, and the gas pressure at the inlet of the high-temperature fixture was 0.01 MPa with a gas flow rate of 8 m^3^/h. The angle between the flow direction of the cooling air through the sample and that through the film hole was 30°, as shown in Figure 5d. Verification tests of the simplified load spectrum method were carried out by comparing the original load spectrum and the simplified load spectrum test results of the flat specimens with single holes. After the verification tests of the simplified load spectrum method, low-cycle creep–fatigue tests were conducted on the flat specimens with multiple holes and circular tube specimens with multiple holes based on the simplified load spectrum.

## 4. Results and Discussion

### 4.1. Analysis of Load Spectrum Verification Test Results

**Figure 7:**
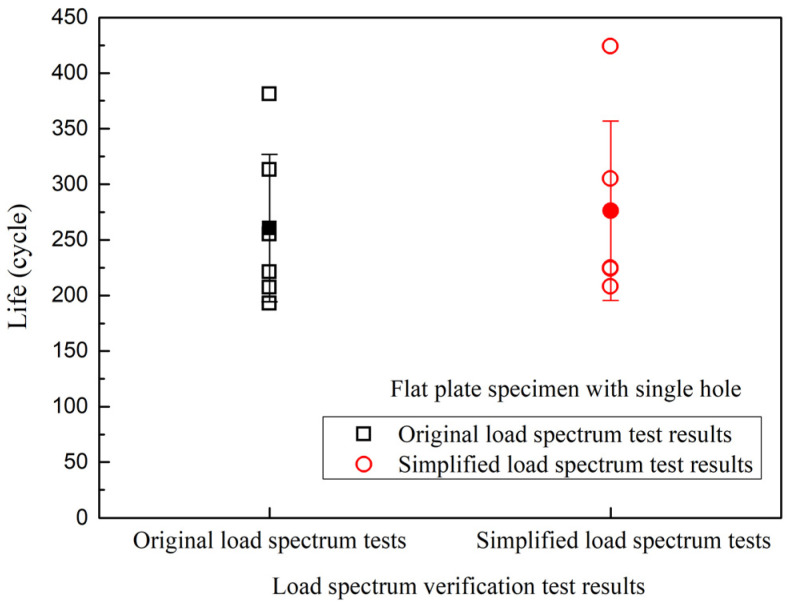
Load spectrum verification test results of flat specimens with single holes.

The low-cycle creep–fatigue test results of the original load spectrum and simplified load spectrum of the flat-plate specimens with single holes are shown in Figure 7. The average value of the original spectrum test results was 261 cycles, with a standard deviation of 67 cycles. The simplified load spectrum test results were slightly higher, with an average value of 277 cycles and a standard deviation of 81 cycles. Compared with the original load spectrum, the simplified load spectrum showed an average life error of 6.13% in the low-cycle creep–fatigue tests of flat-plate specimens with single holes.

**Figure 8:**
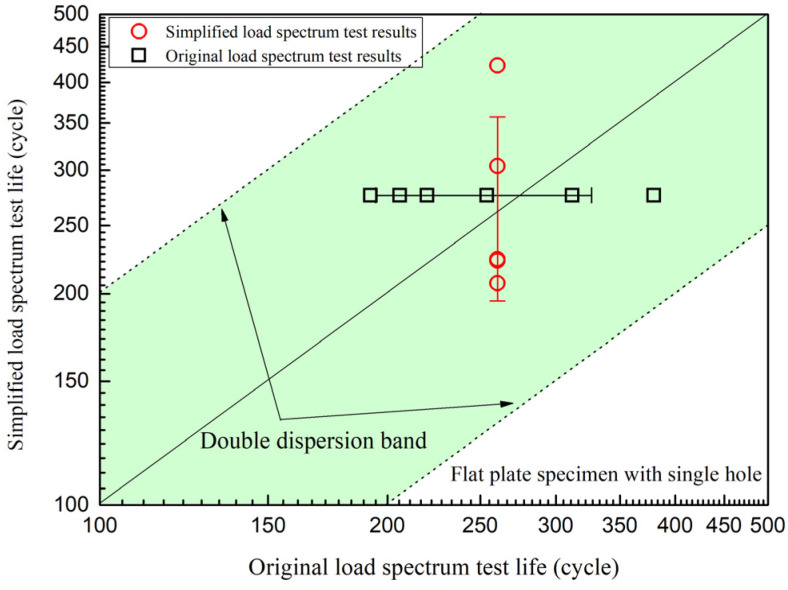
Test results of simplified load spectrum and original load spectrum.

Figure 8 shows the test results of the simplified load spectrum and original load spectrum, with the horizontal axis of the original load spectrum test results and the vertical axis of the simplified load spectrum test results. The simplified load spectrum test results and the original load spectrum test results were both within the double dispersion band of their average values.

### 4.2. Prediction of Low-Cycle Creep–Fatigue Test Life

The fatigue life *N_f_* of GH3230 superalloy with only loading and unloading processes of temperature and stress during each triangular wave cycle was 2173 cycles with a standard deviation of 132 cycles on the thermomechanical fatigue testing of smooth flat-plate specimens. Thus, the fatigue damage *D*_fatigue_ during a single flight mission cycle was 4.60 × 10^−4^. Therefore, the total creep–fatigue damage *D*_total_ during a single flight mission cycle was 2.015 × 10^−3^.

**Figure 9:**
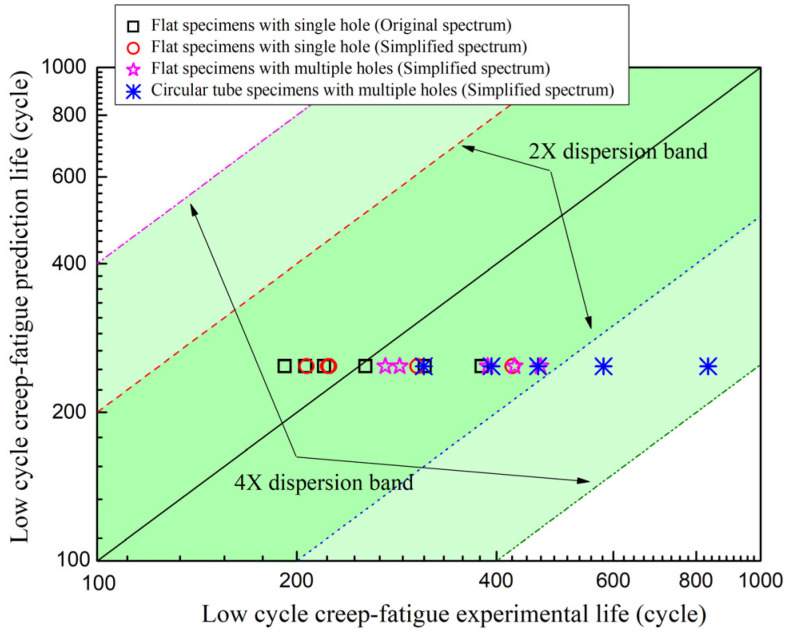
Predicted and experimental results of low-cycle creep–fatigue life.

Based on the creep–fatigue damage assessment method of GH3230 superalloy under multi-stage flight conditions, the creep–fatigue prediction life of the flat specimen with single or multiple holes and circular tube specimen with multiple holes was obtained. The predicted life under nominal stress without holes was 1/*D*_total_ = 496 cycles, while the life with holes can be considered half of the predicted life under nominal stress, which was 248 cycles. The predicted and experimental results of the low-cycle creep–fatigue tests are shown in Figure 9. The experimental results of the flat specimens with single hole or multiple holes were both within the double dispersion band of the predicted results. While the experimental results of the circular tube specimens with multiple holes were basically within the fourfold dispersion band of the predicted results, the average value of experimental results for the circular tube specimens with multiple holes was significantly larger than the predicted results. This indicated that the introduction of internal cooling gas flow reduced the temperature near the gas film holes of the circular tube specimens with multiple holes, thereby improving their low-cycle creep–fatigue test life.

It should be pointed out that this life prediction method may lose accuracy in certain situations: (1) there is an active cooling effect, e.g., there was a cooling airflow inside the circular tube sample, which reduced the temperature near the film holes and significantly increased the actual life, resulting in lower prediction results; (2) the geometric shape is complex or there is high stress concentration; (3) the temperature distribution of the sample is uneven, with a high temperature gradient present; and (4) material behavior deviates from benchmark data.

In addition, it was found that active cooling is one of the most effective ways to improve life. Optimizing the cooling structure and distribution of cooling airflow can effectively improve the lifespan of the combustion chamber while meeting performance requirements. It is necessary to conduct experimental verification from characteristic components to simulated components, which would be helpful for establishing a life-assessment method applicable to specific components.

### 4.3. Temperature Simulation Analysis of Circular Tube Specimens with Multiple Holes

**Figure 10:**
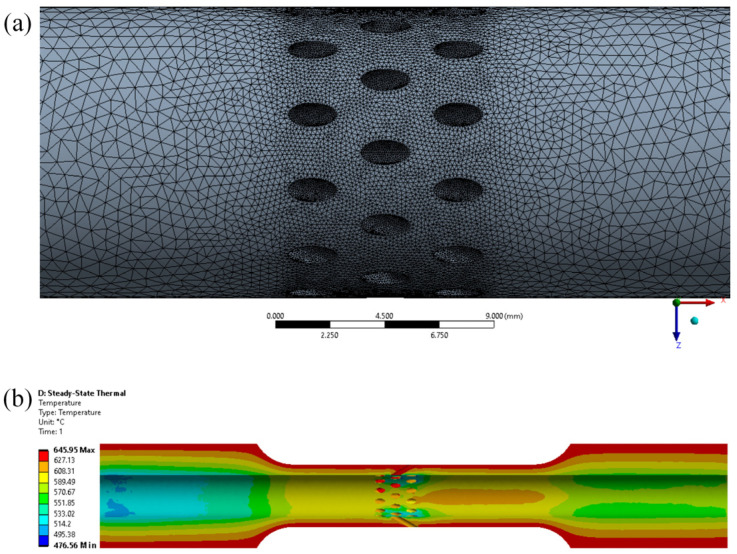
Temperature simulation analysis of circular tube specimen with multiple holes. (**a**) Mesh of the sample; (**b**) temperature field distribution of a cross section of the circular tube specimen with multiple holes.

In order to obtain the temperature around the gas film holes of the circular tube specimens with multiple holes, the temperature field distribution of the circular tube specimens with multiple holes under the peak condition (i.e., the takeoff condition) of low-cycle creep–fatigue testing with cooling gas was calculated using Ansys Fluent 2019 R1 software, as shown in Figure 10. The mesh of the circular tube specimens with multiple holes and tetrahedral mesh is shown in Figure 10a. The unit size at the inclined holes was not greater than 0.1 mm. The specimen’s interior was cooled with room-temperature gas at a pressure of 0.01 MPa and a gas flow rate of 8 m^3^/h, while an external temperature field of 645 °C was applied on the specimen’s outer surface. The mechanical and thermal properties of 3230 superalloy employed are shown in Table 2. The temperature distribution of the circular tube specimen with multiple holes is shown in Figure 10b. The results indicated that under the cooling gas, there was a temperature gradient from the outer surface temperature of 645 °C to the inner surface temperature of 570 °C in the circular tube. The cooling gas was discharged outward from the inclined hole inside the circular tube, and the inclined hole wall temperature was about 590 °C, which was lower than the circular tube outer wall and the inclined hole outside.

### 4.4. Uncertainty Analysis of Circular Tube Specimens with Multiple Holes

In order to assess the uncertainty of the fatigue–creep life prediction results of the circular tube specimens with multiple holes, an uncertainty analysis method based on the error propagation law was used to quantify how the measurement uncertainty of key input parameters, such as temperature, affected the uncertainty of life prediction. This method provided a quantitative basis for understanding the confidence interval of the prediction results, evaluating the applicability of the model and defining the engineering safety margin.

According to the law of error propagation, the uncertainty of the predicted value of LMP can be expressed as a function of the uncertainty of the input value. Thus, the uncertainty of the logarithm of predicted life *u*(lg(*t*)) can be derived as Equation (6).

(6) $$u\left(\mathrm{lg}\left(t\right)\right)=\sqrt{b^{2}u{\left(T\right)}^{2}}=\left|b\right|\cdot u\left(T\right)=bu\left(T\right)$$

According to the simulation results, the inner surface temperature of the circular tube specimens with multiple holes decreased to 570 °C, with a maximum temperature gradient of 75 °C (i.e., Δ*T* = 75 °C = 135 °F). For uniform distribution, the standard temperature uncertainty *u*(*T*) can be obtained by converting the half-width of uniformly distributed intervals into standard uncertainty.

(7) $$u\left(T\right)=\frac{135{}^{\circ}F}{\sqrt{3}}\approx 77.94{}^{\circ}F$$

Thus:

(8) $$u\left(\mathrm{lg}\left(t\right)\right)\approx 0.855$$

This means that at a confidence level of about 68%, the true fatigue–creep life lg(t) value will fluctuate within the range of its estimated value of 2.394–3.249. Therefore, when the inner surface temperature of the circular tube specimens with multiple holes decreases by 75 °C, the estimated fatigue–creep life may be extended to $10^{0.855}\approx 7.16$ times, which is consistent with the experimental results in Figure 9.

### 4.5. Fracture Analysis

**Figure 11:**
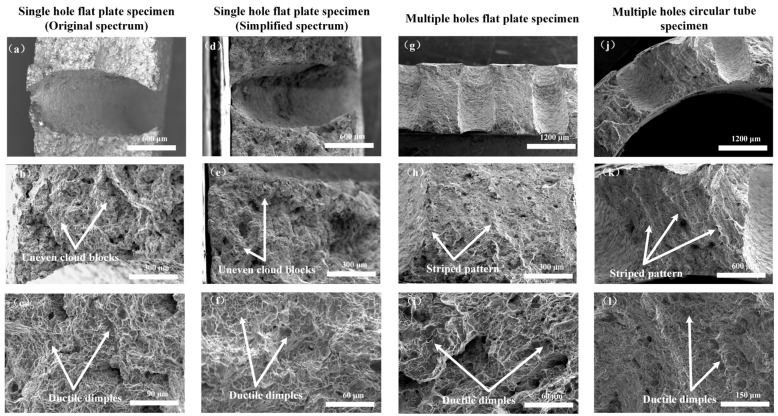
Fracture analysis of low-cycle creep–fatigue test specimen. (**a**,**b**,**c**) Single hole flat plate specimen under original spectrum; (**d**,**e**,**f**) single hole flat plate specimen under simplified spectrum; (**g**,**h**,**i**) multiple holes flat plate specimen; (**j**,**k**,**l**) multiple holes circular tube specimen.

After low-cycle creep–fatigue testing, the fracture surfaces of the specimens were cut, placed in acetone and ethanol solutions for ultrasonic cleaning, and dried. The samples were then observed using a scanning electron microscope (SEM, FEI Quanta FEG 250, Hillsboro, OR, USA). The results showed that all specimens exhibited ductile fracture under creep–fatigue interaction, with microscopic features dominated by dimples, and microvoid coalescence, consistent with the typical fracture morphology of nickel-based superalloys under high-stress loading. The fracture surfaces of the flat specimens with single holes under the original load spectrum are shown in Figure 11a–c. Figure 11b displays the region near the hole edge, which appears as uneven cloud-like blocks, reflecting grain boundary sliding and creep void coalescence due to local high-stress concentration, which is a typical characteristic of creep damage. Figure 11c shows dense dimples in the ductile fracture tearing region, indicating the high plastic deformation capability of GH3230 superalloy. The fracture surfaces of the flat specimens with single holes under the simplified load spectrum are shown in Figure 11d–f, with morphology similar to that of the original loading spectrum specimens. The fracture surfaces of the flat and circular tube specimens with multiple holes are shown in Figure 11g–i and Figure 11j–l, respectively. Figure 11h,k depicts regions near the hole edges, where the fracture surfaces appear striated with irregular tearing ridges, indicating that the multi-hole structure exacerbated the multiaxial stress state and promoted a tendency toward brittle fracture. Figure 11i,l shows the ductile tearing dimple regions. The dimple distribution in the flat and circular tube specimens with multiple holes was uneven, indicating that inter-hole interference led to strain localization and accelerated creep damage.

In addition, laser machining of holes is an effective and commonly used method. However, microcracks and heat-affected zones occur in the hole wall [25,26]. When laser-machining holes, the high temperature at the edge of the hole will cause nonuniform temperature distribution and plastic strain, which may make it easier for cracks to initiate and propagate from the hole edge in low-cycle creep–fatigue tests.

## 5. Conclusions

Based on the damage equivalence principle, the low-cycle creep–fatigue original load spectrum of a combustion chamber was simplified, which included multi-stage flight conditions, such as low speed, takeoff, climb, and cruise states. The simplified method was validated through flat specimens with single holes. Furthermore, low-cycle creep–fatigue life predictions were conducted for the feature simulation specimens of the combustion chamber, including flat specimens with single or multiple holes and circular tube specimen with multiple holes. The conclusions are as follows.

(1) Compared with the original load spectrum, the simplified load spectrum showed an average life error of 6.13% in the low-cycle creep–fatigue tests of flat-plate specimens with single holes.
(2) The simplified load spectrum test results and the original load spectrum test results were both within the double dispersion band of their average values.
(3) The low-cycle creep–fatigue test results of the flat specimens with single or multiple holes were both within the double dispersion band of the predicted results, while the test results of the circular tube specimens with multiple holes were basically within the fourfold dispersion band of the predicted results.
(4) The introduction of internal cooling gas flow reduced the temperature near the gas film holes of the circular tube specimens with multiple holes, thereby improving their low-cycle creep–fatigue test life.

## Figures and Tables

**Table 1:** Chemical composition of GH3230 superalloy (wt.%) [3,7,23].

Elements	C	Cr	Ni	Co	W	Mo	Al	Ti
Mass fraction/%	0.05–0.15	20.00–24.00	balance	≤5.00	13.00–15.00	1.00–3.00	0.20–0.50	≤0.10
Elements	Fe	La	B	Si	Mn	S	P	Co
Mass fraction/%	≤3.00	0.005–0.05	≤0.015	0.25–0.75	0.30–1.00	≤0.015	≤0.05	≤0.50

**Table 2:** Basic mechanical properties of GH3230 superalloy [3,7,23].

Temperature/°C	Elasticity Modulus *E*/GPa	Poisson Ratio *v*	Yield Strength/MPa	Tensile Strength/MPa	Thermal Expansion Coefficient α/10^−6^ °C^−1^	Thermal Conductivity *λ*/W/(m·K)	Specific Heat Capacity *c*/J/(kg·K)	Thermal Diffusivity *Q*/10^−6^ m^2^/s
25	215	0.31	389.5	905–910	/	12.3	/	/
200	204	0.31	/	/	13.5	14.2	469	2.95
400	194	0.32	/	/	14.4	16.7	503	3.57
600	182	0.32	316.6	735–740	14.7	20.1	530	4.06
700	176	0.33	289.9	620–635	15.3	22.4	/	/
800	168	0.33	234.2	405–410	15.7	25.04	550	4.71
900	160	0.34	142.7	250–285	16.0	27.2	/	/
1000	150	0.35	71.5	151–157	16.3	29.3	534	4.67

**Table 3:** Creep endurance properties of GH3230 superalloy [23].

Temperature/°C	Stress/MPa	Life/h	Temperature/°C	Stress/MPa	Life/h
1000	42	26.2	800	160	49.2, 52
	33	66.6		153	44.5, 49.5
	23	220.4		133	142.6
	16	1583.9		120	309
900	75	17.6, 18		100	693
	62	50.6, 51.3	750	200	74.8, 91.8
	55	88, 102.8		170	270
	45	443.9		150	689
	40	747.6		130	2104.8
870	114	18, 23.8	700	310	38.2, 78.5
	90	37.5, 45.3		260	106.3, 125.2
	83	103, 106.5		220	288.3
	72	228.2, 285.3		180	1304.2
	60	832.5			

**Table 4:** Original load spectrum for dangerous combustion chamber position in single flight mission cycle.

Service Time /min	Service Condition	Dangerous Position Stress/MPa	Dangerous Position Temperature/°C
0–15	Low speed	10	190
15–18	Takeoff	414	645
18–173	Climb	329	592
173–196	Cruise	203	524
196–199	Low speed	10	190

**Table 5:** Takeoff condition damage converted by total creep damage during a single flight mission cycle.

Flight Conditions	Service Life/h	Damage	Total Damage *D*_totel_ During a Single Flight Mission Cycle	Converted Takeoff Condition Time/min
Low speed	>10^7^	0	1.555 × 10^−3^	5.5
Take off	59.125	8.46 × 10^−4^		
Climb	3642.2	7.09 × 10^−4^		
Cruise	2,303,525.4	1.66 × 10^−7^		

**Table 6:** Low-cycle creep–fatigue tests.

Specimens	Original Spectrum Tests	Simplified Spectrum Tests	Thermomechanical Fatigue Tests
Smooth flat specimen	/	/	5
Flat specimen with single hole	6	5	/
Flat specimen with multiple holes	/	5	/
Circular tube specimen with multiple holes	/	5	/

## Data Availability

The original contributions presented in this study are included in the article. Further inquiries can be directed to the corresponding author.

## References

[1] Zheng Q.A., Cai C.P., Zhang H.W., Zhang H.B. (2024). Prediction of engine combustion chamber outlet temperature field based on deep learning: Application in aero-engine life extension control. Appl. Therm. Eng..

[2] Wang Z., Wang Z.P., Ding K.Y., Dai H.W. (2024). Failure analysis and reliability assessment of a type of aero-engine combustion chamber thermal barrier coatings. Case Stud. Therm. Eng..

[3] Wang C.J., Qiao Z.Y., Tao H.Y., Xu P.Y., Liu H.B., Li X.T. (2025). The scale-adaptive simulation of reverse coupling of aero-engine high pressure high-pressure turbine to combustion chamber flow. Appl. Therm. Eng..

[4] Hu L., Wang W.T., Wang X., Yang J.G., Yu Y.H., Mei C.Y. (2025). Reliability evaluation and multi-objective optimization of combustion chamber’s key components of marine engine. Case Stud. Therm. Eng..

[5] Zhang Y.J., Hou C., Jin X.C., Li D., Zhao L., Yang L., Fan X. (2024). Thermal fatigue crack initiation and propagation behaviors of GH3230 nickel-based superalloy. Fatigue Fract. Eng. Mater. Struct..

[6] Xu Q.P., Hou C., Ren X.D., Yang J., Jin X., Yang Q., Fan X. (2025). Study on creep behaviors of GH3230 superalloy with side notches or a center inclined hole at 900 °C and 1000 °C. Fatigue Fract. Eng. Mater. Struct..

[7] Zhao H., Cheng D., Chen M., Xiao W., Hou C. (2025). Research on Creep Behaviors of GH3230 Superalloy Sheets with Side Notches. Materials.

[8] Zhao L., Wang X., Xu L.Y., Han Y.D., Jing H.Y. (2021). Fatigue performance of Hastelloy X at elevated temperature via small punch fatigue test. Theor. Appl. Fract. Mech..

[9] Esmaeilzadeh M., Qods F., Arabi H., Sadeghi B.M. (2017). Evolution of the texture and microstructure in a nickel based superalloy during thermo-mechanical fatigue (TMF), using a modified integrated model and experimental results. Int. J. Fatigue.

[10] Healey R., Wang J., Wallbrink C., Chiu W.K., Xu Z. (2022). The application of cycle merging and an extension of a fatigue spectrum simplification methodology from unidirectional to woven composite materials. Compos. Part C Open Access.

[11] Healey R., Wang J., Chiu W.K., Chowdhury N.M., Baker A., Wallbrink C. (2021). A Review on Aircraft Spectra Simplification Techniques for Composite Structures. Compos. Part C Open Access.

[12] Moucun Y., Hong N. (2007). Analysis approach to durability based on material initial fatigue quality and S-N curve. Chin. J. Aeronaut..

[13] Vueti N., Jovii G., Krsti B., Ivkovi M., Milovanovi V., Kamarik J., Antunovi R. (2020). Research of an aircraft engine cylinder assembly integrity assessment—Thermomechanical FEM analysis. Eng. Fail. Anal..

[14] Molent L., Singh R. (2019). Using the lead crack framework to reduce durability test duration. Aeronaut. J..

[15] Liu X.N., Tan J.H., Long S.B. (2024). Multi-axis fatigue load spectrum editing for automotive components using generalized S-transform. Int. J. Fatigue.

[16] Fang J., Li K.Y., Liu C.X., Zou S., Sun B. (2024). A novel two-scale nonlinear damage accumulation model for vibration fatigue life prediction. Int. J. Non-Linear Mech..

[17] Zheng G.F., Duan Y.L., Su H., Liu X.L., Xu X.Y., Hao H. (2025). Novel method for accelerated multi-axial load spectrum editing with phase preservation based on the fusion of damage and energy domain features. Eng. Fract. Mech..

[18] Chen L., Ding K.Q. (2024). Research on multiaxial fatigue life prediction of lifting equipment based on symbol corrected equivalent stress spectrum. J. Phys. Conf. Ser..

[19] Jiang R.J., Yang X.G., Cheng M.W., Huang J., Shi D.Q. (2025). Experimental investigation and life prediction for the load spectrum with flight mission characteristics on a p/m superalloy. Chin. J. Aeronaut..

[20] He X.F., Dong Y.M., Zhai B., Liu W.T. (2011). The fleet life reliability analysis under the 90% severe load spectrum. Eng. Fail. Anal..

[21] He X.F., Sui F.C., Dong Y.M., Liu W.T. (2010). Relative severity investigation in a severe load spectrum. Eng. Fail. Anal..

[22] Li X., Sun Q. (2021). Simplification approaches for multi-level load spectra by using equivalent damage rule. Chin. J. Aeronaut..

[23] Zhang J., Sun X.F., Li J.R., Dong J.X. (2012). China Superalloys Handbook.

[24] Wen Z.X., Zhang D.X., Zhang Z.J., Zhang W., Li L., Wang X.M., Yue Z.F. (2017). Structural Strength of Film Cooling Holes in Nickel-Based Single-Crystal Turbine Blades.

[25] Du C.Y., Cui H.T., Zhang H.J. (2024). Thermal fatigue behaviors of thin-walled structures with holes: Experiments and phase field fracture modeling. Int. J. Fatigue.

[26] Liang J.S., Qiao H.C., Zhao J.B., Cao Z.H., Zhang Y.T., Zhang Q. (2025). Simulation and experimental study on double staggered-axis air-assisted water jet-guided laser film cooling hole machining. Opt. Laser Technol..

